# Simultaneous production of fresh water and electricity via multistage solar photovoltaic membrane distillation

**DOI:** 10.1038/s41467-019-10817-6

**Published:** 2019-07-09

**Authors:** Wenbin Wang, Yusuf Shi, Chenlin Zhang, Seunghyun Hong, Le Shi, Jian Chang, Renyuan Li, Yong Jin, Chisiang Ong, Sifei Zhuo, Peng Wang

**Affiliations:** 1 Water Desalination and Reuse Center, Division of Biological and Environmental Science and Engineering, King Abdullah University of Science and Technology, Thuwal, 23955-6900 Saudi Arabia; 2 KAUST Solar Center (KSC), King Abdullah University of Science and Technology, Thuwal, 23955-6900 Saudi Arabia

**Keywords:** Energy harvesting, Energy storage

## Abstract

The energy shortage and clean water scarcity are two key challenges for global sustainable development. Near half of the total global water withdrawals is consumed by power generation plants while water desalination consumes lots of electricity. Here, we demonstrate a photovoltaics-membrane distillation (PV-MD) device that can stably produce clean water (>1.64 kg·m^−2^·h^−1^) from seawater while simultaneously having uncompromised electricity generation performance (>11%) under one Sun irradiation. Its high clean water production rate is realized by constructing multi stage membrane distillation (MSMD) device at the backside of the solar cell to recycle the latent heat of water vapor condensation in each distillation stage. This composite device can significantly reduce capital investment costs by sharing the same land and the same mounting system and thus represents a potential possibility to transform an electricity power plant from otherwise a water consumer to a fresh water producer.

## Abstract (web summary)

The increasing demand for energy and clean water has become a grand global challenge. Here the authors develop a membrane-distillation device that exploits sunlight and the heat dissipated by an integrated solar cell unit, enabling simultaneous efficient production of electricity and drinkable water.

## Introduction

Water and energy are inextricably linked and the intimate water-energy nexus is being increasingly felt globally, as water security is becoming a threat to energy security and vice versa^1,2^. In the United States and Western Europe, about 50% of water withdrawals are for energy production^3,4^. On the other hand, clean water production, especially seawater desalination, consumes huge amount of electricity. In Arab countries, for example, more than 15% of the total national electricity is consumed by fresh water production industry^5^. It has been reported that 1~10% of clean water produced in electricity-driven seawater desalination process is fed back to power plant to generate the electricity consumed during desalination process^6,7^. The ramifications of the water-energy nexus have been greatly aggravated especially in arid and semi-arid regions.

The current share of nonrenewable fossil fuels in the global energy mix is still larger than 82% and the burning of fossil fuels leads to a massive CO_2_ emission, which is regarded as a major threat to the global sustainability^8^. Great effort has been made to adopt renewable energy sources, among which solar energy has shown its immense potential to meet the world’s future energy demands given its vast abundance and free availability. Huge amount of photovoltaics (PV) panels (>400 GW) have been installed all over the world to generate electricity from solar energy with minimal CO_2_ emission and water consumption. For 1 MWh electricity generation, PV technology consumes only 2 gallons of water while thermal power plants using coal and nuclear fuel as energy source consume 692 and 572 gallons of water, respectively^9^. However, solar irradiation has a quite low energy intensity, generally in the range of 4–8 kW m^−2^ per day for the most parts of the world^10^. Moreover, only about 10–20% of the energy from sunlight can be converted to electricity by the state-of-the-art commercial PV panels^11^. As a result, for a medium-sized solar power plant of 400 MW, it would need to collect sunlight from at least an entire 2 million m^2^ land area. Besides the cost of the solar panels and land procurement, mounting system supporting the panels on such a large area adds further capital cost of solar power plant^11^.

Solar distillation has recently attracted considerable attention and has demonstrated promising potentials in various processes aimed at seawater desalination^12–20^, potable water production from quality-impaired water sources^21,22^, wastewater volume reduction^20^, metal extraction and recycling^23^, and sterilization^24^, etc. However, similar to the solar-to-electricity conversion, the inherent low energy intensity of solar irradiation leads to a small fresh water production rate in conventional solar distillation, 0.5–4.0 kg m^−2^ in a whole day, equivalent to water production rate of 0.3–0.7 kg m^−2^ h^−1^ under the standard one Sun illumination condition (1 kW m^−2^)^16,25,26^. The low productivity necessitates large land area and installation of mounting system to support distillation setup, which constrains its economic benefit, similar to the case of PV power plants. Very recently, solar-driven multistage membrane distillation (MSMD) devices have been reported with a much higher clean water productivity, 3 kg m^−2^ h^−1^ in a 10-stage device under one Sun illumination, by recycling the latent heat released during vapor condensation in each stage as the heat source for the next stage^27,28^.

The concept of simultaneous production of clean water and electricity by solar energy has been recently investigated by several groups^29–31^. In most of these attempts, solar distillation was utilized for clean water production and some side effects of the solar distillation were utilized for electricity generation, which led to low solar-to-electricity energy efficiency (<1.3%). The low electricity generation efficiency of these strategies makes it uneconomical to apply them in commercial power plant.

In this work, we report a strategy for simultaneous production of fresh water and electricity by an integrated solar PV panel-membrane distillation (PV-MD) device in which a PV panel is employed as both photovoltaic component for electricity generation and photothermal component for clean water production. In a typical solar cell, 80–90% of the absorbed solar energy is undesirably converted to heat, and thereafter passively and wastefully dumped into the ambient air^32^. In this work, a MSMD device is integrated on the backside of a commercial solar cell to directly utilize its waste heat as a heat source to drive water distillation. Under one Sun illumination, the water production rate of the PV-MD is 1.79 kg m^−2^ h^−1^ for a 3-stage device, which is three times higher than that of the conventional solar stills. At the same time, the PV panel generates electricity with energy efficiency higher than 11%, which is the same as that recorded on the same PV panel without the back MD device and which is at least 9 times higher than those achieved in the previously published works. The undoubted benefit of the integration of PV and water distillation is the highly efficient co-generation of clean water and electricity in one device at the same time on the same land, which directly reduces land area requirement and the cost of the mounting system as compared to two physically separate systems (PV and solar distillation). Moreover, working directly with commercial solar cells makes the PV-MD device close to practical applications. This strategy provides a potential possibility to transform an electricity generation plant from otherwise a water consumer to a fresh water producer.

## Results

### Structure of the MSMD device

The solar cell harvests short wavelength sunlight to generate electricity via photovoltaic effect, which results in a high solar-to-electricity energy efficiency. Large amount of waste heat is simultaneously generated as a side effect during electricity generation from two pathways. The first one is the relaxation of short-wavelength sunlight excited electrons and the second pathway is photothermal conversion of the long-wavelength sunlight. The waste heat is regarded as a burden in conventional solar power plants and is directly dumped into ambient air as waste. In our design, the heat is considered as resource and is delicately utilized as energy source to power PV-MD device to produce clean water.

**Fig. 1:**
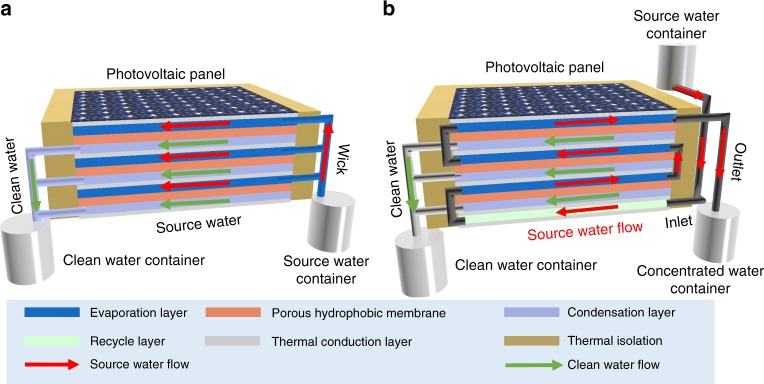
Schematic illustration of the integrated photovoltaics-membrane distillation (PV-MD) devices. Operate in **a** dead-end mode (in this mode, the source water is wicked into the evaporation layer in the direction of the red arrow and the condensed water flows out from the condensation layer in the direction of the green arrow) and **b** cross-flow mode (in this mode, the source water flows to the evaporation layer in the direction of the red arrow and the condensed water flows out from the condensation layer in the direction of the green arrow)

In this work, a commercial polycrystalline silicon solar cell from Sharp was adopted both as electricity generation component and photothermal component. A lab-made MSMD device was constructed on the backside of the solar cell for clean water production (Fig. 1a and Supplementary Fig. 1). In order to reduce heat loss into the ambient environment, the sides of the PV-MD device were sealed by polyurethane (PU) foam with low thermal conductivity (0.022~0.033 W m^−1^ K^−1^)^33^.

Each stage of the MSMD device was composed of four separate layers: a top thermal conduction layer, a hydrophilic porous layer of water evaporation layer, a hydrophobic porous layer of MD membrane for vapor permeation, and a water vapor condensation layer. Aluminum nitride (AlN) plate was used as the thermal conduction layer because of its extremely high thermal conductivity (>160 W m^−1^ K^−1^) and its anti-corrosion property in salty water^34^. The hydrophobic porous layer was made of an electrospun porous polystyrene (PS) membrane. The water evaporation layer and condensation layer were of the same material, a commercial hydrophilic quartz glass fibrous (QGF) membrane with non-woven fabric structure.

In each stage of the MSMD device (Supplementary Fig. 2), the heat is conducted through the thermal conduction layer to the underlying hydrophilic porous layer. The source water inside the hydrophilic porous layer is thus heated up to produce water vapor. The water vapor passes through the hydrophobic porous membrane layer and ultimately condenses on the condensation layer to produce liquid clean water. The driving force for the water evaporation and vapor condensation is the vapor pressure difference caused by the temperature gradient between the evaporation and condensation layers. In each stage, the latent heat of water vapor, which is released during the condensation process, is utilized as the heat source to drive water evaporation in the next stage. The multistage design ensures the heat can be repeatedly reused to drive multiple water evaporation–condensation cycles. In a traditional solar still, the heat generated from the sunlight via photothermal effect only drives one water evaporation–condensation cycle, which sets up an upper theoretical ceiling of the clean water production rate, ~1.60 kg m^2^ h^−1^, under one-Sun condition in such a system. The multistage design makes possible to break the theoretical limit as demonstrated very recently by two groups^27,28^.

In this work, two source water flow modes, namely, dead-end mode and cross-flow mode, are designed for the MSMD device (Fig. 1). In the dead-end mode, the source water is passively wicked into the evaporation layer by hydrophilic quartz glass fibrous membrane strips via capillary effect. In this case, the concentration of salts and other non-volatile matters in the evaporation layer keeps increasing till reaching saturation in the end. A washing operation is indispensable to remove the salts accumulated inside the device for this mode, as reported in the previous works^28^. However, the passive water flow reduces the complexity of the device and gives a high water production rate in the early stage for this operation mode. In the cross-flow mode, the source water flows into the device driven by gravity or by a mechanical pump, and, it flows out of the device before reaching saturation. In this case, the outgoing water flow will take away a small amount of sensible heat, leading to a slight drop in clean water productivity in the early stage. However, it solves the salt accumulation problem and avoids the need for frequent cleaning and salt removal operation, which makes the device suitable for long-term operation.

In some experiments, a commercial spectrally selective absorber (SSA) (ETA@Al, Alanod Solar) was used to replace the PV panel for clean water production performance evaluation. This material can decrease the radiation heat loss during operation because it possesses a much smaller emissivity than PV panels, and that is why it was adopted in both of the previous works on solar membrane distillation^28^. We use the SSA-MD device to confirm that the multistage MD device we fabricated in this work is comparable with the state-of-the-art solar membrane distillation devices.

### Solar absorptance of the SSA and photovoltaic cell

**Fig. 2:**
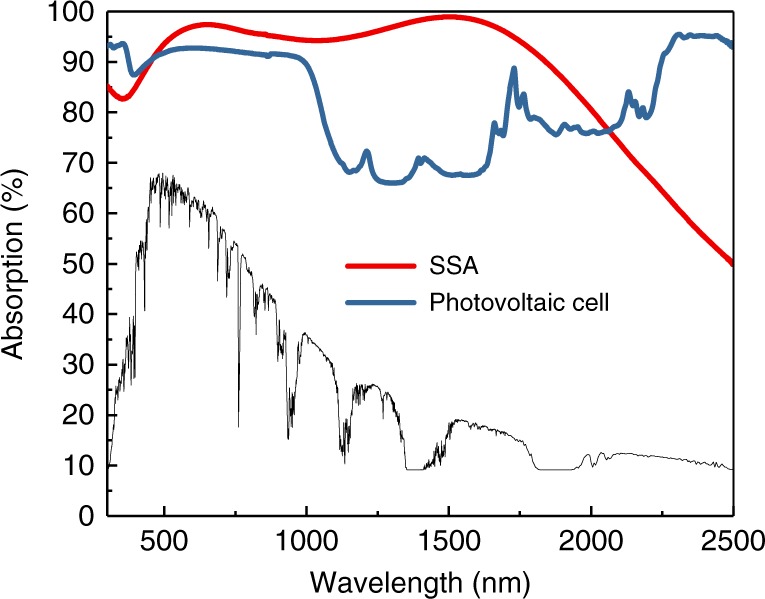
UV-Vis-NIR spectra of the solar cell and the spectrally selective absorber (SSA) material. (The standard solar radiation spectra of air mass 1.5 global (AM 1.5G) is shown by the black line.)

The UV-Vis-NIR absorption spectrum of the solar cell was collected and presented in Fig. 2. As seen, the solar cell possesses a high light absorption (>92%) in short wavelength range (<1000 nm) and a slightly lower absorption (70–80%) in long wavelength range. Since the solar spectrum is not uniformly distributed, solar absorptance (α), which is defined as a weighted fraction between absorbed radiation energy and incoming solar radiation energy, is calculated to estimate the solar energy capture ability of the solar cell by the following equation^35^:

1 $$\alpha = \frac{{\mathop {\smallint }\nolimits_{300}^{2500} I(\lambda )A(\lambda )d\lambda }}{{\mathop {\smallint }\nolimits_{300}^{2500} I\left( \lambda \right)d\lambda }}$$

Where *I*(λ) and *A*(λ) represent the light intensity and absorption of a material at different wavelength. The solar absorptance of the solar cell used in this work is calculated to be 0.87, indicating 87% solar energy is harvested by the solar cell. The thermal emissivity of the solar cell is evaluated to be 0.930 (Supplementary Fig. 3 and Supplementary Note 1). It has been reported that most commercial solar cells possess high light absorption and high emissivity because they are designed to capture as much sunlight as possible and dump the waste heat as fast as possible^36,37^. In comparison, the commercial SSA material shows efficient absorption (>95%) in short wavelength region (<1600 nm) and good reflectance in long wavelength region (>1600 nm), which is the characteristic of SSA type materials. The solar absorptance and emissivity of the SSA material are 0.94 and 0.123, respectively, which are similar to those reported in literatures^38,39^.

### Clean water and electricity production performance

**Fig. 3:**
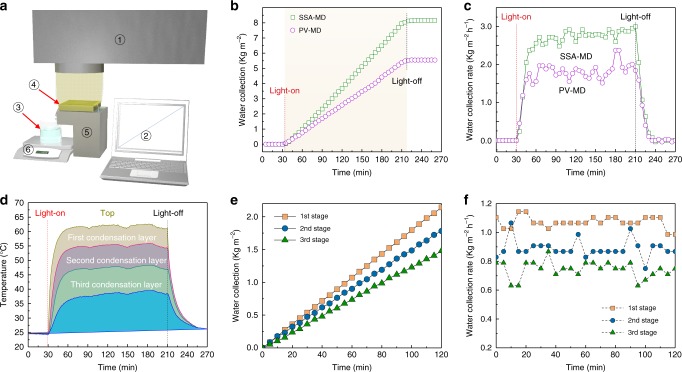
Water production evaluation of the multistage membrane distillation (MSMD) device. **a** Schematic representation of the experimental setup (① Solar simulator, ② computer ③, clean water collector, ④ photovoltaics/spectrally selective absorber-membrane distillation (PV-MD/SSA-MD), ⑤ source water container, ⑥ electrical balance). **b** The mass change rates of the collected water under one sun irradiation (starting from the red dash line) and dark (starting from the black dash line), and **c** water production rates of a three-stage dead-end PV-MD/SSA-MD devices, **d** temperature profile, **e** the mass change of the collected water and **f** the water production rate of each stage of the three-stage dead-end SSA-MD device

The clean water production performance of a multistage SSA-MD device operated in dead-end mode was firstly evaluated in a lab-made setup (Fig. 3a), with pure water as the source water. The average water production rate, calculated from the slope of the mass change curve at the steady state (Fig. 3b), was 2.78 kg m^−2^ h^−1^ for 3-stage SSA-MD and was 3.25 kg m^−2^ h^−1^ for 5-stage SSA-MD (Supplementary Fig. 4), which is about 5 times the fresh water production rate of the state-of-the-art conventional solar stills. The multistage MD device fabricated in this work is comparable to the state-of-the-art multistage MD devices^27,28^.

When the SSA material was replaced with the solar cell as photothermal component in the 3-stage PV-MD device and the PV-MD was not connected to an external circuit, i.e. the solar cell was used just as a photothermal material and the absorbed solar energy was converted to heat exclusively without any electricity output, the average water production rate was 1.96 kg m^−2^ h^−1^ (Fig. 3c), which is 29.5% lower than that of the 3-stage SSA-MD device. This significant decrease of clean water production for PV-MD can be attributed to its slightly lower solar energy harvesting and much more radiation heat loss, which will be discussed later.

For SSA-MD with 3-stage structure, after reaching the steady state, the temperature of the conduction layer from top to bottom was 61.8, 55.1, 47.5 and 38.4 °C (Fig. 3d). The corresponding temperature difference between the top surface of the water evaporation layer and bottom surface of the condensation layers in the 1st, 2nd and 3rd stage of the SSA-MD device was 6.7 °C, 7.6 °C and 9.1 °C, respectively. A MD stage working at higher temperature gives higher energy utilization efficiency as reported in numerous literatures^40–42^.

The water production rate in the 1st, 2nd and 3rd stage of the 3 stage SSA-MD was 1.07, 0.89, and 0.75 kg·m^−2^·h^−1^, respectively (Fig. 3e, f). The water production rates of the 2nd and 3rd stage were equivalent to 83% and 84% of the 1st and 2nd stages, respectively, indicating a high latent heat recovery rate. It should be pointed out that this result does not mean that only ~83% of the latent heat was recycled by the next MD stage and the rest was lost. Actually, since the device was well sealed by the PU foam in all side surface, the heat loss through the side surface is negligible, and therefore the heat flux in all these three MD stages is almost the same. The decrease in clean water production rate is mainly because of the lower working temperature in the 2nd and 3r^d^ stages, which led to a lower clean water production efficiency.

**Fig. 4:**
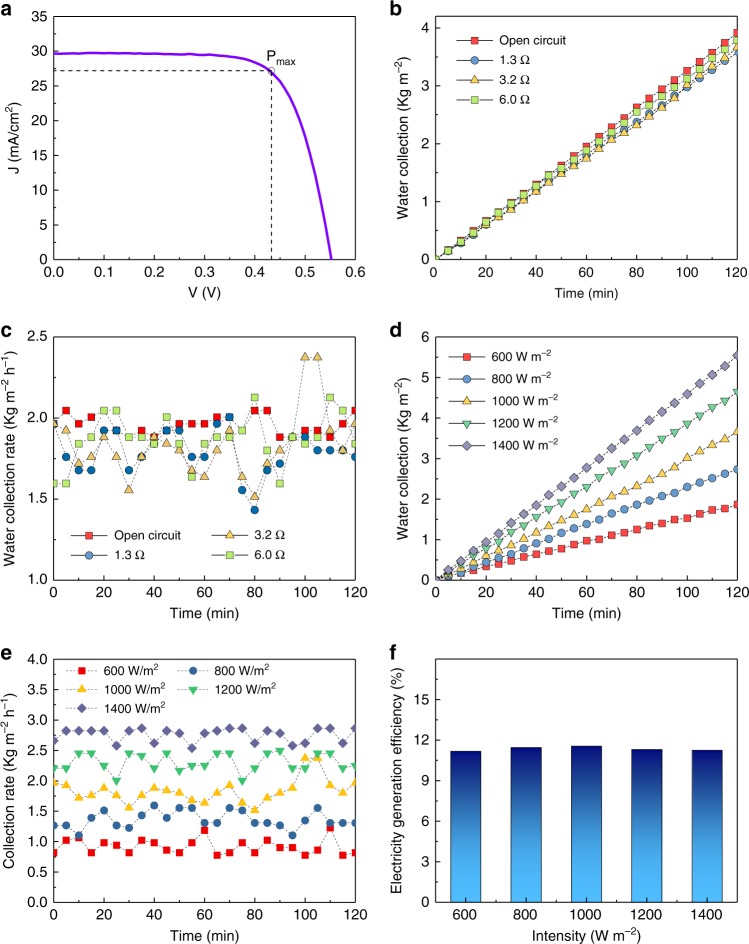
Electricity and water production evaluation of the photovoltaics-membrane distillation (PV-MD) device. **a** J–V curve of the solar cell under one Sun illumination (*P*_max_ refers to the maximum power). **b** The mass change rate of the collected water and **c** clean water production rate at different loads of 3-stage PV-MD with dead-end mode; **d** the mass change rate of the collected water, **e** clean water production rate, and **f** electricity generation efficiency under different solar irradiation intensity of 3-stage PV-MD with dead-end mode

The water production performance of the 3-stage PV-MD was next further investigated by connecting the solar cell to an external circuit with different resistances. When the device was working under one-Sun illumination with pure water as source water, the temperature of the solar cell, which is slightly affected by the external resistance, was measured to be approximately 58 °C. Since the performance of the solar cell is affected by its working state temperature, the J–V curve of the solar cell at working state (58 °C) was measured under one-Sun illumination condition with simultaneous clean water and electricity production operation (Fig. 4a). Based on the J–V curve, the largest output power was 138 mW for this solar cell, which was achieved under an optimal load of 1.3 Ω with a current of 0.32 A and output voltage of 0.43 V. Although the effective working area of the MSMD device (4.0 cm × 4.0 cm) was 16 cm^2^, the effective working area for the solar cell was only 11.9 cm^2^ (Supplementary Fig. 5). The energy efficiency of the solar cell under this condition was calculated to be 11.6%.

When the solar cell was connected to a resistance with its optimal load (1.3 Ω), the same PV-MD exhibited a water production rate of 1.79 kg m^−2^ h^−1^ (Fig. 4b, c), which is 8.7% lower than that without electricity output. When the resistance of the load was increased to 3.2 and 6.0 Ω, the output power was decreased to 84 and 50 mW, with an increase of output voltage to 0.52 and 0.53 V, respectively. The water production rates were 1.82 and 1.88 kg m^−2^ h^−1^ for these two cases, respectively (Fig. 4b, c). These results indicate that the water production rate is only slightly affected by the extraction of electricity from the system, which is expected. Overall, the device gave a high clean water productivity ( > 1.79 kg m^−2^ h^−1^) given that about 11% solar energy was extracted from the PV-MD device to produce electricity.

The clean water production performance of the 3-stage dead-end PV-MD device under solar illumination with different light intensity was also investigated and the results are presented in Fig. 4d–f. The average water production rates under 0.6, 0.8, 1.0, 1.2, and 1.4 Sun illumination were measured to be 0.92, 1.39, 1.82, 2.31, and 2.65 kg m^−2^ h^−1^, respectively (Fig. 4d, e). The relationship between the clean water production rate and solar irradiation intensity was linear (Supplementary Fig. 6) and the electricity generation efficiency of the solar cell was stable at around 11.1~11.6% under different solar irradiation. These results demonstrate that the PV-MD device possesses excellent clean water production and stable electricity generation performance under varying solar intensity.

**Fig. 5:**
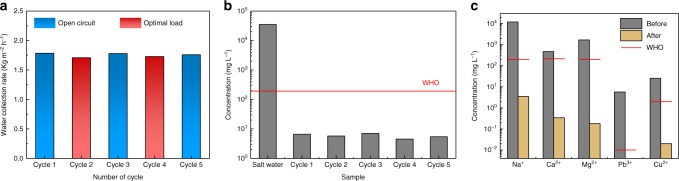
Reusability evaluation of the photovoltaics-membrane distillation (PV-MD) device. **a** Water production rate in different cycles under open circuit state (blue column) and optimal stage (red column) via the 3-stage photovoltaics-membrane distillation (PV-MD) device with dead-end mode for salt water desalination, **b** water salinity of the source water and desalinated water collected in every cycle (The red line is the World Health Organization’s (WHO) guidelines for drinking-water quality). **c** Ion concentrations of the heavy-metal contaminated source water and desalinated water by PV-MD device

One targeted application of PV-MD is to generate electricity and at the same time produce clean water from various source water with impaired quality, such as seawater, brackish water, contaminated surface water, and groundwater. When 3.5% NaCl aqueous solution was used as a seawater surrogate, the clean water production rate was 1.77 kg m^−2^ h^−1^ in open circuit state and 1.71 kg m^−2^ h^−1^ in the optimal load state (1.3 Ω). These two values are both lower than those recorded when pure water was used as source water (Fig. 4), which should be attributed to the decrease of the saturation vapor pressure of the salt water^35^. For the devices operated at dead-end mode, the salt concentration of the source water in the evaporation layer would gradually increase during operation, leading to a slight decrease in clean water production rate (Supplementary Fig. 7). The concentrated source water inside the device can be sucked out of the device by a dry paper via capillary effect. Although not all the NaCl salt was removed in this way, the performance of the device could be nearly fully recovered in the next operation cycle. Figure 5a shows the clean water production rate of the dead-end device measured in five operation cycles. In cycle 1, 3, and 5, the solar cell was not connected to external circuit, while in cycle 2 and 4, the solar cell was connected to external circuit (Fig. 5a). The result clearly demonstrates that this device can be regenerated from salt accumulation state with fully recovered performance. The concentration of Na^+^ in the collected condensate water in each cycle was always lower than 7 ppm, which is only 0.02% of the source water and much lower than the World Health Organization (WHO) drinking water standard (Fig. 5b). In another experiment, PV-MD with dead-end mode was used to produce clean water from a heavy metal-contaminated seawater. The PV-MD device exhibited a clean water production rate of 1.69 kg m^−2^ h^−1^ under one-Sun illumination (Supplementary Fig. 7). The concentrations of the ions in the source water and clean water product were measured and shown in Fig. 5c. For the collected clean water, the concentrations of Na^+^, Ca^2+^, and Mg^2+^ decreased to be lower than 4 ppm while the concentrations of Pb^3+^ and Cu^2+^ decreased to almost zero and 0.02 ppm, respectively. All of the ion concentrations are below the WHO drinking water standards^43^. These results convincingly indicate a perfect desalination performance via the membrane distillation process.

In a PV-MD device operated at dead-end mode, the salts from the source water will continuously accumulate inside the evaporation layer during operation as mentioned above, which may cause failure and damage if salt crystals block the pores of the MD membrane. Although the salt can be cleaned out of the device by frequent regeneration operation as discussed earlier, it deems not practical for long-term operation and large-scale application. Therefore, we further designed a 3-stage PV-MD device that can be operated at cross-flow mode to solve the salt accumulation problem (Fig. 1b). In this device, a source water flow layer (recycle layer) was added at the bottom part to recycle the heat for the purpose of pre-heating the source water before it enters into the evaporation layer. When the water outlet of this 3-stage cross-flow type PV-MD device was blocked, i.e., it was operated in a dead-end mode with no water flowing out of the device, the clean water production rate was 2.09 kg·m^−2^ h^−1^ (Supplementary Fig. 8) with pure water as source water, which is 7% higher than that recorded on the dead-end type device under the otherwise same conditions (1.96 kg·m^−2^ h^−1^) (Fig. 1a). This result suggests that adding a source water flow layer at the bottom to recycle the heat can improve the clean water productivity.

When the water outlet of the 3-stage cross-flow type PV-MD device was opened and the flow rate of the source water was controlled to be 5 g h^−1^, which is about two times the water production rate in the dead-end condition, the clean water production rate was slightly decreased to 1.93 kg m^−2^ h^−1^. This can be explained by the fact that some sensible heat was carried away by the outgoing water flow at the outlet. When the flow rate of the source water was increased to 6 and 7 g h^−1^, the clean water production rates were further decreased to 1.83 and 1.76 kg;m^−2^ h^−1^, respectively. These results indicate that the clean water production rate was only slightly affected by the flow rate of the source water because the outgoing water contains only small amount of sensible heat.

The seawater desalination performance of the 3-stage PV-MD device with cross-flow mode was then evaluated and is presented in Supplementary Fig. 8. The flow rate of the source water was controlled at 5 g h^−1^ to avoid continuous salt accumulation inside the device and the device exhibited a very stable clean water production rate of 1.65 kg m^−2^ h^−1^ under one-Sun illumination in a 3-day continuous test. In this case, a continuous concentrated source water stream steadily flowed out of the device, keeping the salt concentration at a steady state inside the device. The salt concentration of the source and concentrated seawater was 3.8 wt% and 8.7 wt%, respectively. Although the clean water production rate was slightly lower when the device was operated under this condition, comparing to dead-end mode, its long-term clean water production stability outweighs its slightly reduced rate. Field tests of a large PV-MD device were conducted and the details can be found in Supplementary Fig. 9 and Supplementary Note 2.

## Discussion

In the MSMD device, the latent heat released during vapor condensation process in each MD stage is reused as the energy source for the next MD stage. Therefore, the heat generated by the photothermal component is thus reused for multiple evaporation–condensation cycles. As a result, the clean water production rates of both 3-stage SSA-MD and PV-MD, e.g., 2.78 and 1.96 kg m^−2^ h^−1^, are several times higher than that of conventional solar stills and even break the theoretical limit of a perfectly full utilization of solar irradiation of one Sun intensity within the conventional one-stage device. The large clean water production rate is a great advantage of this type of newly developed multistage MD devices comparing to conventional solar stills (Supplementary Table 1).

The energy balance scheme of this PV-MD device is different from the conventional solar stills and conventional membrane distillation devices (Supplementary Fig. 2). When it is exposed under sunlight with intensity of q_S_, most of the solar energy is captured by the solar cell (q_A_) depending on its solar absorptance (α), and the rest is lost as the reflected sunlight. The total energy obtained from sunlight (one Sun condition) by SSA and PV (Sharp) is thus calculated to be 930 and 870 W·m^−2^, respectively. Part of the captured solar energy is converted to electricity (q_e_) for PV-MD, depending of the efficiency of the solar cell (η), which is generally in the range of 10–20% for a commercial solar cell. The rest of the absorbed solar energy is converted to heat (q_h_). Their relation can be described as follows:

2 $${\mathrm{{q}}}_{\mathrm{{A}}} = \alpha \times {\mathrm{{q}}}_{\mathrm{{S}}}$$

3 $${{{\rm{q}}_{\rm{e}}}} = \eta \times {{{\rm{q}}_{\rm{A}}}}$$

4 $${{{\rm{q}}_{\rm{h}}}} = {{{\rm{q}}_{\rm{A}}}} - {{{\rm{q}}_{\rm{e}}}}$$

Some of the heat energy is directly lost from the top surface of the solar cell by thermal radiation, thermal conduction, and air convection. Because of the extremely low thermal conductivity of air (0.01~0.04 W m^−1^ K^−1^), thermal radiation is the main pathway, which can be calculated by Stefan–Bolzmann equation:

5 $$E = \varepsilon \sigma (T_{{\mathrm{{Cell}}}}^4 - T_0^4)$$

Where ε is the emissivity of the material, *σ* is the Stefan–Bolzmann constant, *T*_Cell_ is the temperature of the material, and *T*_0_ is the temperature of its surroundings. The emissivity of the solar cell (Sharp) used in this work is 0.940 (Supplementary Fig. 3 and Supplementary Note 1), which is similar to most commercial solar cells but is much higher than the SSA material used in this work (0.123). When the 3-stage device is exposed under one Sun illumination, the solar cell or the SSA material is quickly heated up to 58.4 and 61.8 °C during operation, respectively. Accordingly, the heat loss from the top surface via thermal radiation is estimated by the Stefan–Bolzmann equation (Eq. (5)) to be 219 W m^−2^ for PV-MD and 33 W m^−2^ for SSA-MD. For the PV-MD, there is also 90 W m^−2^ energy converted to electricity and is therefore extracted from the system. Consequently, the energy power that can be utilized for the MSMD component of PV-MD and SSA-MD in clean water production is 561 W m^−2^ and 907 W m^−2^. This estimation helps us understand why the SSA-MD device gives a much higher clean water production rate than PV-MD device as presented previously. It also clearly reveals that the emissivity of the top surface is the key parameter that affects the heat utilization efficiency of the MSMD device, implying that if the emissivity of the solar cell can be reduced, the clean water production performance may be significantly improved. Actually, the high emissivity of the commercial solar cells is an intentional design^36,37^, because it promotes the waste heat discharge from the solar panels to their surroundings, and therefore lowers down the temperature of the solar panels. However, it is possible to significantly decrease the emissivity of the solar panels without affecting its solar-to-electricity conversion efficiency^44,45^. In other words, the clean water production rate of PV-MD can be further increased in future by making solar cells with smaller emissivity.

In each MD stage, the heat energy solely comes from its previous stage through the top heat conduction layer. Part of the heat can be lost to the outer environment from the side surfaces of the device, which, however, should only account for a negligible share in this work based on the following considerations. (1) The PU foam on the side surface possesses a very small thermal conductivity (0.022~0.033 W m^−1^ K^−1^) and relatively large thickness (> 1 cm); (2) the area of the side faces is much smaller than the cross-section area of the device, especially when the size of the device is larger than 10 × 10 cm and the height of each stage is only 0.5 cm.

During the membrane distillation process, since the heat loss from side faces is negligible, almost all the heat that comes into the evaporation layer is finally transferred into the condensation layer, and then conducted to the next MD stage. For each MD stage, the heat transfer from the evaporation layer to condensation layer is mainly composed of two pathways. In the first pathway, the heat (Q_I_) is transferred as the latent heat of water vapor through the water evaporation–condensation process. This is a simultaneous heat transfer and mass transfer process. The mass transfer process in this pathway is responsible for the clean water production. In the second pathway, the rest of the heat (Q_II_) in the evaporation layer is directly transferred to the condensation layer via thermal conduction without any mass transfer. Only the first pathway contributes to the clean water production and the second pathway is considered as a waste of thermal energy. The heat utilization efficiency (η_heat_) can be defined as the percentage of the total heat transferred through the first pathway.

6 $${{\eta_{{\rm{heat}}}}} = \frac{{{\mathrm{Q}}_{\mathrm{{I}}}}}{{{\mathrm{{Q}}}_{\mathrm{{I}}} + {\mathrm{{Q}}}_{{\mathrm{{II}}}}}}$$

The total heat transfer flow of the 3-stage SSA-MD device is estimated to be 907 W m^−2^ as discussed above. The heat flow by the first pathway (Q_I_) can be estimated from the clean water production rate for each stage following the Eq. (7).

7 $$Q_I = m \times L_V$$

Where m refers to the clean water production rate, *L*_*v*_ refers to the latent heat of the water evaporation, which is calculated following an empirical formula from literature^20^. As mentioned before, the clean water production rate of the 1st, 2nd and 3rd stage in the 3-stage SSA-MD device is 1.07, 0.89, and 0.75 kg m^−2^ h^−1^ and the working temperature of each stage is calculated to be approximately 58, 51 and 43 °C (Fig. 3d). Accordingly, the heat flux in the first pathway (Q_I_) for the 1st, 2nd and 3rd stage are 710, 595, and 506 W m^−2^, equivalent to heat utilization efficiency (η_heat_) of 78%, 66%, and 56%, respectively.

In another word, although the same amount of the heat energy (907 W m^−2^) is transferred from the evaporation layer to condensation layer at all stages, assuming the heat loss through side surface is zero, higher portion of energy is transferred through the first pathway when the MD stage is working at a higher temperature, which agrees with literatures^32,41,42^. On the other hand, more heat is transferred through the second pathway in the lower stages working at lower temperatures. The heat flux through the second pathway in the 1st, 2nd and 3rd stages is estimated to be 197, 312 and 401 W m^−2^, respectively. Since the thermal conductivity of the MD membrane is only slightly affected by the temperature, a larger heat flux through the second pathway corresponds to a larger temperature difference between top and bottom of the MD stage, which is corroborated with the results recorded in this work. The temperature difference between the top and bottom thermal conduction layer for the 1st, 2nd and 3rd stage in the 3-stage SSA-MD device is 6.7, 7.6 and 9.1 °C, respectively (Fig. 3d).

A simplified thermal model is developed for MSMD (details can be found in the Supplementary Note 3 and Supplementary Figs. 10 and 11). According to the model, if more MD stages are further added to the PV-MD devices, the clean water production rate can be certainly improved. However, the temperature of the solar cell will increase in response, which would cause increased thermal radiation heat loss from the top surface of the solar cell. Consequently, the thermal energy that can be utilized by the MSMD component will decrease, i.e., the heat flux that transfers through each stage is decreased. As a result, although the total clean water production rate can be increased, the efficiency in each stage will decrease, which raises up the relative cost of the device. In addition, a higher operation temperature will decrease the energy efficiency of the solar cell and its lifetime. Therefore, the number of the stages in these devices should be limited by a rational value after thorough consideration of various factors.

The global PV capacity is expected to increase to 969 GW by 2025^37^, which need approximately 4 billion m^2^ land area to collect sunlight. Assuming that PV-MD devices with 3 stages are to be installed on these lands, there are 200 days each year with suitable solar irradiation for the PV-MD to operate and the PV-MD has a conservative water production rate of 5.0 kg m^−2^ day^−1^, there will be approximately 4 billion cubic meter (m^3^) fresh water produced every year from all kind of source waters along with the required and expected electricity production by the PV-MD devices. This is equivalent to 10% of the total global drinking water consumption in 2017^39^. The generated clean water can be used for many highly desired and niche purposes, such as cleaning solar panel to remove dust particles, irrigation of plants and crops and drinking water especially in semi-arid and arid areas, potable water production from highly contaminated surface and groundwater, etc. The PV-MD device thus has potential to transform the conventional power plant from a huge water consumer to an electricity plus clean water co-producer and to make a meaningful contribution to the currently very stressed water-energy nexus (see more details in Supplementary Note 4).

## Methods

### Materials

QGF membrane was purchased from Merck Millipore (catalog number AQFA8X105), polystyrene (PS, *M*_w_ = 280,000 g/mol) and *N,N*’-dimethylformamide (DMF) were purchased from Sigma-Aldrich. The solar cell of 3.9 × 3.9 was purchased from Sharp and the solar cell of 12 × 12 cm size was purchased from SunPower. Spectra selective absorber ETA@Al was purchased from Alanod Solar. Polyurethane (PU) foam system containing part A and part B was purchased from Aldon Corporation.

### Synthesis of the polystyrene membrane

The hydrophobic polystyrene membrane was fabricated by electrospinning method. Polystyrene was firstly dissolved in DMF by mild stirring for 6 h to obtain 25 *wt*% homogeneous solution. The solution was placed in three 5-mL syringes equipped with metal needle of 0.52 mm inner diameter and then ejected with a feeding rate of 5.0 mL h^−1^. The voltage was set at 30 KV and the distance between the collector and the needle was 10 cm.

### Device assembly

The QGF, PS membrane and AlN were assembled as shown in Supplementary Fig. 1a. To avoid blocking the wick of the dead-end device by PU foam, the wick was firstly wrapped by plastic film. The PU foam precursor was obtained by mixing the part A and part B of the PU system as 1:1 weight ratio and then was painted on the side of the device. The device was kept at 50 °C for 12 h to complete the foaming. The assembly of cross-flow MSMD was similar to that of the dead-end MSMD, except that the wick was replaced with the silicon tube with the diameter of 1 mm.

### Simultaneous production of clean water and electricity

The device was put on the top of a square heat sink with a length of 5 cm which was immersed in bulk water. Water was transported from bulk water to the device by capillary effect and transpiration effect via a small QGF membrane belt which was connected to the distillation layer. In a practical scenario, the flow rate of the source water in the cross-flow PV-MD can be controlled by a flow control valve or flow meter. In our experiments, an ISMATEC tubing pump was used to control the flow rate of the source water more precisely. Solar irradiation was provided by a solar simulator (Newport 94043A) with a standard AM 1.5 G spectrum optical filter. A 100 mL cup was used to collect clean water and the amount of water collected was monitored and recorded real-time. To reduce the evaporation of the collected clean water in the cup, a funnel was put on the top of the cup. The square photothermal material or square solar cell with the length of 3.9 mm was put on the top of the device. Photovoltaic responses (J–V curves) of the solar cell were measured by a Keithley 2400 series source meter. For the cycling test, after each cycle, 3 pieces of Kimwipes tissue (11 × 21 cm) were connected to the inlets of the dead-end devices and stayed there for 3 h to extract the concentrated water remaining in the QGF membranes. For each new cycle, salt water was wicked into the QGF membrane and extracted by the tissue again. This procedure was repeated at least for 3 times to ensure that the QGFs were cleaned.

### Characterization

The UV-Vis-NIR diffuse reflectance spectra of the samples were recorded with an Agilent Cary 5000 spectrometer, with BaSO_4_ powder as reference. The concentrations of the ions in water were measured by Inductively Coupled Plasma Optical Emission Spectrometer (ICP-OES). The emissivity was measured by using a FLIR A655 infrared camera as follows: the solar cell and SSA were put on a heating plate and a thermal couple was used to measure the temperatures of them. After they were heated to a designated temperature, the infrared camera was used to measure the temperature and the emissivity of the camera software was adjusted to make the temperature of the infrared camera to match the temperature of the thermal couple.

## Supplementary information

Supplementary Information

## Data Availability

The data that support the findings of this study are available from the corresponding authors on reasonable request.
