# Supplementary material for: Simultaneous production of fresh water and electricity via multistage solar photovoltaic membrane distillation
Source: Nat Commun. 2019 Jul 9;10:3012. doi: 10.1038/s41467-019-10817-6 (PMC6616361; doi:10.1038/s41467-019-10817-6)
Supplement: Supplementary file 1 — Supplementary Information [file 41467_2019_10817_MOESM1_ESM.pdf]

Simultaneous Production of Fresh water and Electricity via Multistage Solar Photovoltaic Membrane Distillation

Wenbin et al.

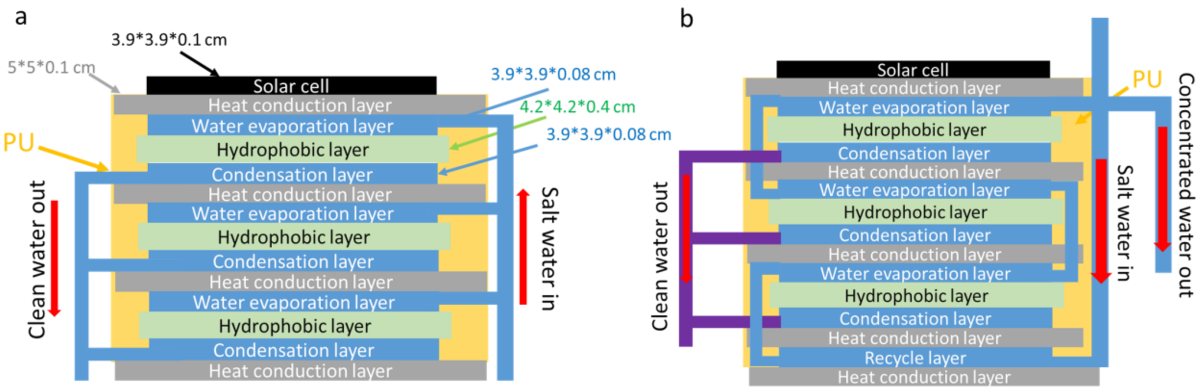

Supplementary Figure 1 Structure of the 3-stage photovoltaics-membrane distillation (PV-MD) device. Operate at **a** dead-end mode and **b** cross flow mode.

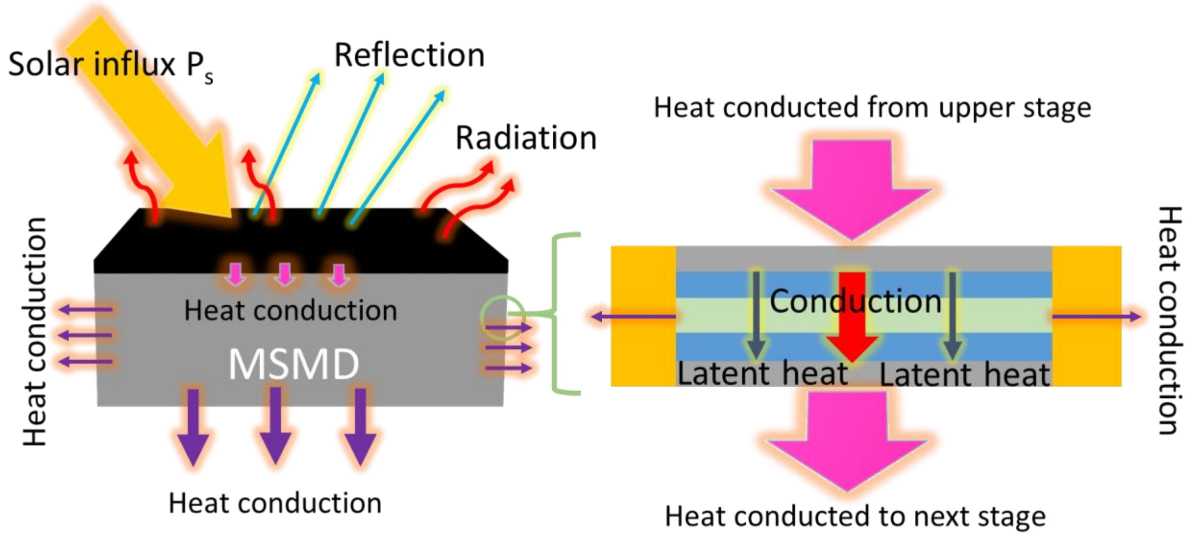

Supplementary Figure 2 Energy balance diagram of a multistage membrane distillation (MSMD) device (left) and a single stage (right).

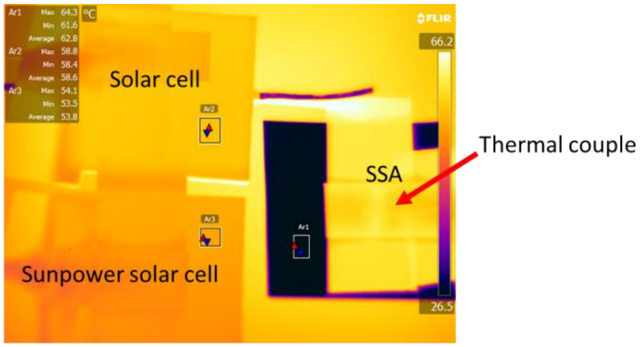

Name	Infrared temperature	Thermal couple temperature	Emissivity
SSA	62.82	62.65	0.123
Solar cell[1]	58.61	58.46	0.930
Solar cell[2]	53.82	54.99	0.995

Supplementary Figure 3 IR image of the spectrally selective absorber (SSA) and solar cell and the results of the emissivity measurement. (Solar cell[1] and Solar cell[2] are from Sharp and Sunpower, respectively.)

Supplementary Note 1

The emissivity was measured by using a FLIR A655 infrared camera as follows: the solar cell and SSA were put on a heating plate and a thermal couple was used to measure the temperatures of them. After they were heated to a designated temperature, the infrared camera was used to measure the temperature. The emissivity of the camera software was adjusted to make the temperature of the infrared camera to match the temperature of the thermal couple.

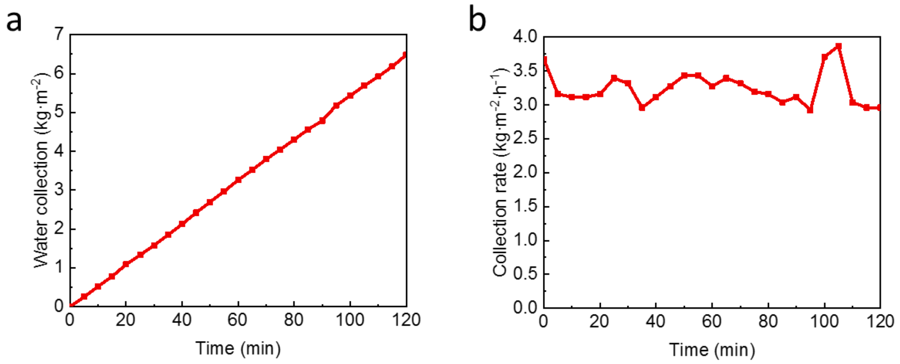

Supplementary Figure 4 Clean water production performance of 5-stage dead-end mode SSA-MD. **a** Mass change of the collected water and **b** clean water production rate as a function of time.

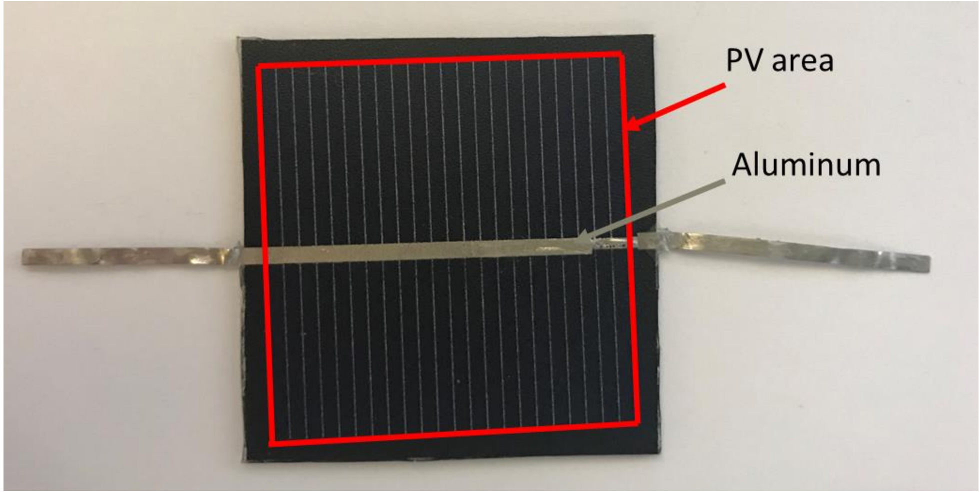

Supplementary Figure 5 Photo image of the solar cell (Sharp).

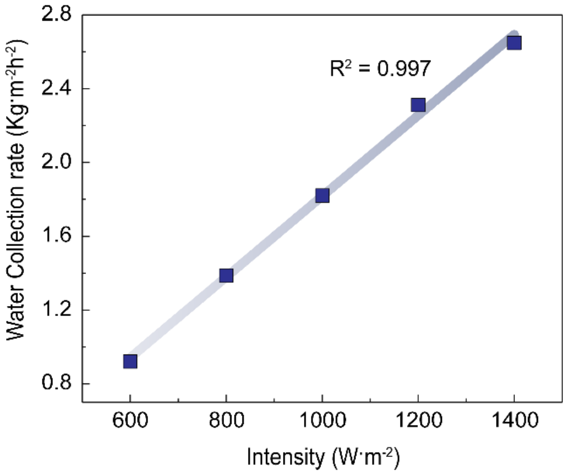

Supplementary Figure 6 Linear correlation of the clean water production rate and solar irradiation intensity of 3-stage photovoltaics-membrane distillation (PV-MD) in dead-end mode

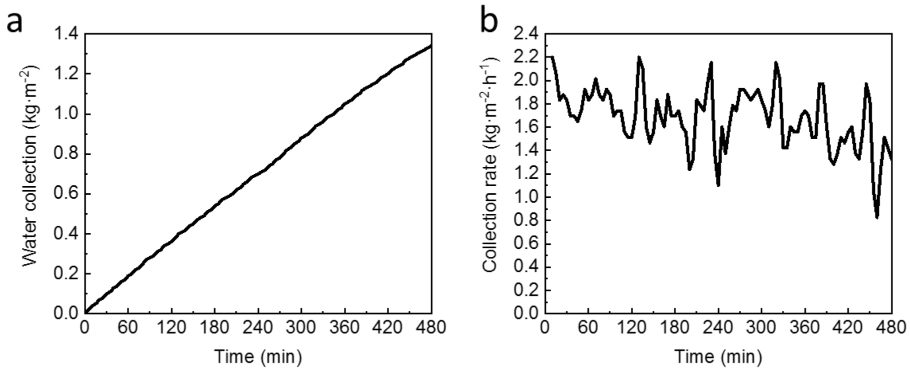

Supplementary Figure 7 Clean water production from seawater contaminated by heavy meatal in the 3-stage dead-end mode PV-MD. **a** Mass change of the collected water and **b** clean water production rate as a function of time.

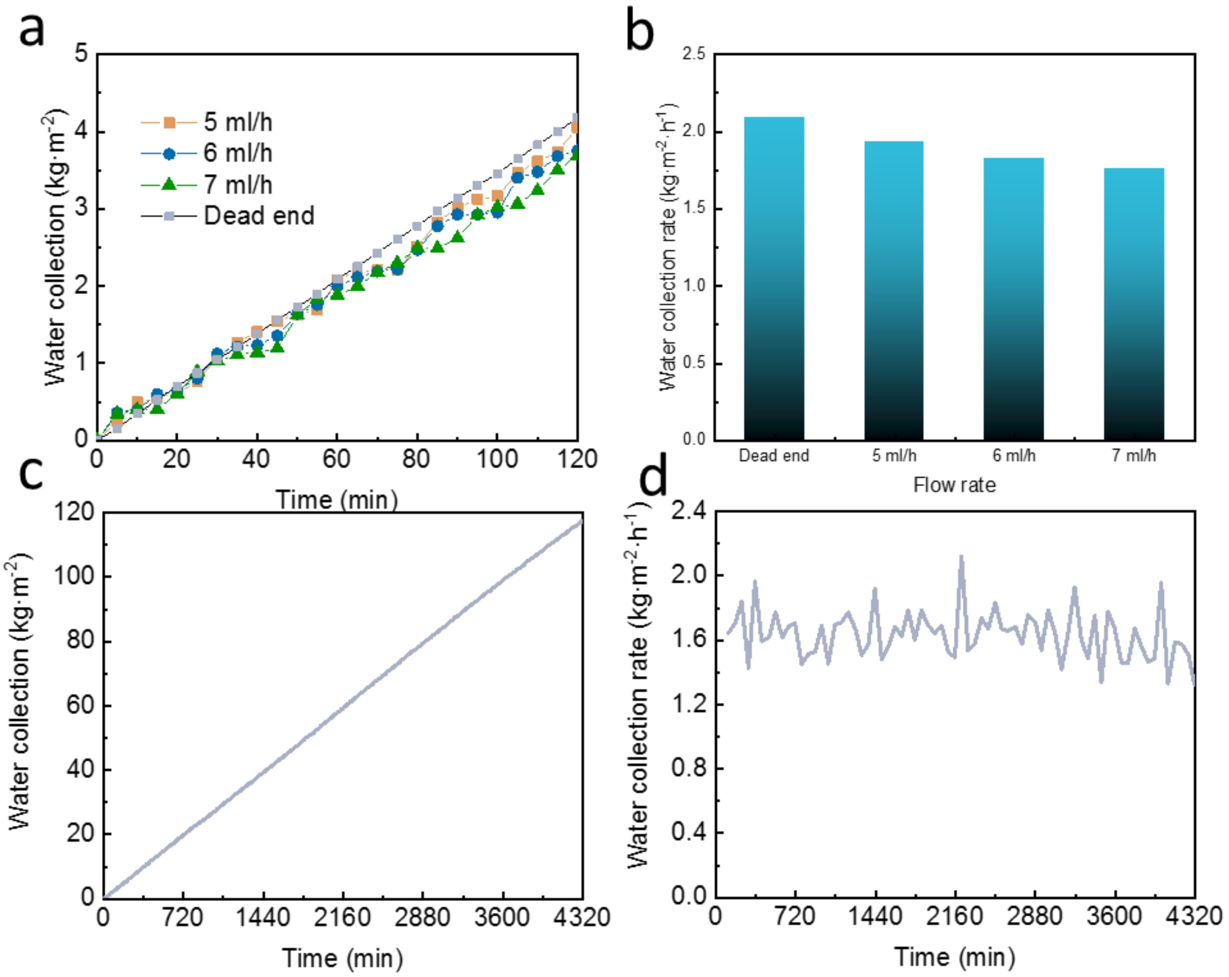

Supplementary Figure 8 Clean water production performance of the 3-stage cross-flow mode PV-MD device. **a** The mass change rates of the collected water, **b** water production rates as a function of water flow rate, **c** the mass change rates of the collected water, and **d** water production rates as a function of time.

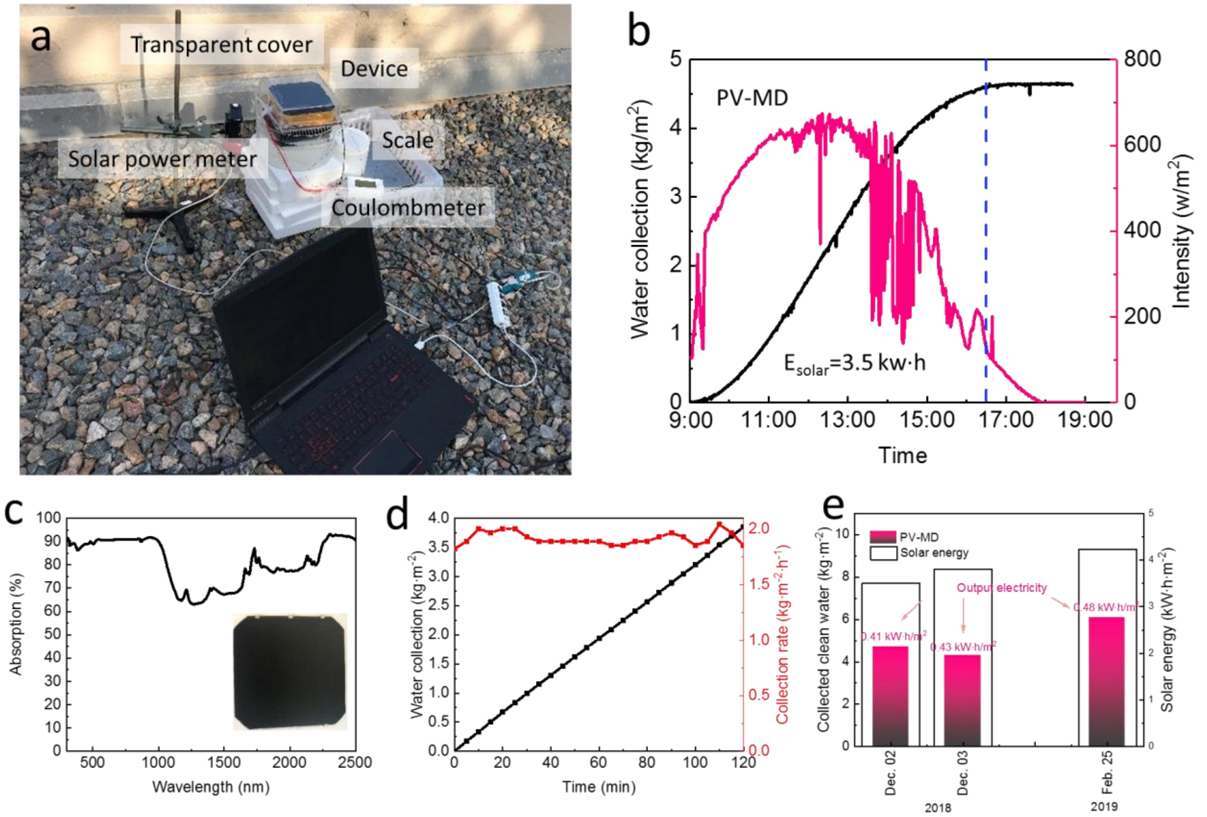

Supplementary Figure 9 The outdoor test condition and performance. **a** the photo of the outdoor test setup, **b** mass change of the collected water of the dead-end mode 5-stage photovoltaics-membrane distillation (PV-MD) and the solar power intensity curve, **c** UV-Vis-FTIR spectra of the solar cell (SunPower), **d** clean water production performance of the solar cell (SunPower) in the 3-stage PV-MD device, **e** the water production and electricity generation performance of the dead-end mode 5-stage PV-MD in the outdoor test on December 02, 2018, December 03, 2018, and February 25, 2019.

Supplementary Note 2

A larger 5-stage dead-end PV-MD device (10 cm × 10 cm) was fabricated with a monocrystalline silicon solar cell from SunPower and the outdoor test was conducted (Supplementary Figure 9). The solar absorptance of the solar cell was measured to be 0.86 (Supplementary Figure 9c), which is a bit lower than the previous solar cell. According to the manual of the solar cell, the maximum electricity generation efficiency of the solar cell was 22.7% and the voltage and the current at optimal load condition was 0.58 V and 6.0 A, implying its maximal output power of 3.48 W and its optimal load being very small (i.e., 0.097 Ω). It should be mentioned that in large scale applications, several to tens of solar cells are installed in series to provide a high output voltage, *e.g.*, 5 or 20 V, where the optimal load can be within a more common resistance range. In this work, only one solar cell was used in our device owing to the size limit of the PV-MD device and the external load was 0.25 Ω based on facility availability, meaning the solar cell was not working in its optimal condition. The device was examined for its clean water and electricity production performance outdoors on the balcony of a student apartment inside KAUST campus, Thuwal, KSA (22°N 39°E) in winter (December 2, 2018) (Supplementary Figure 9a and 9b). The balcony was exposed to daily sunlight only after 9:00 am. Therefore, the experiment was carried out from 9:00 am to 19:00 pm. The total solar irradiance in this period was measured by a solar intensity meter and calculated to be 3.5 $kW{\cdot}m^{-2}$ per day, which is a relatively low value due to the winter season condition. In summer, this value can be tripled. A transparent cover made of poly(methyl methacrylate) (PMMA) was put on the top of the device to reduce the heat loss caused by the strong wind in that day. The final total clean water production was 4.7 $kg{\cdot}m^{-2}$ and the total output electricity energy was measured by a coulombmeter to be 0.41 $kW{\cdot}m^{-2}$. Given the total solar energy of 3.5 $kW{\cdot}m^{-2}$ in this test period, the power conversion efficiency of the solar cell was calculated to be 11.7%.

The outdoor clean water production and electricity generation performance of the devices was also evaluated on February 25, 2019 with a stronger sunlight intensity and the results are shown in Supplementary Figure 9e. The total solar irradiance in the testing period (i.e., 9:00 am to 19:00 pm) was 4.2 $kW{\cdot}m^{-2}$. The large 5-stage dead-end PV-MD device delivered a clean water production rate of 6.1 $kg{\cdot}m^{-2}$ per day and a total output electricity energy of 0.50 $kW{\cdot}h{\cdot}m^{-2}$ with a power conversion efficiency of 11.9%. As discussed earlier, the power conversion efficiency of the solar cell is significantly dependent on the load of the external circuit, the reduced power conversion efficiency was caused by the high resistivity of the load (0.25 Ω), which is much higher than the optimal load (0.097 Ω). Furthermore, as can be seen in Supplementary Figure 9b, when the intensity of the sunlight was below 180 W/m^2, the clean water production rate was nearly zero, which may be ascribed to the extremely low temperature gradient in the device under this condition.

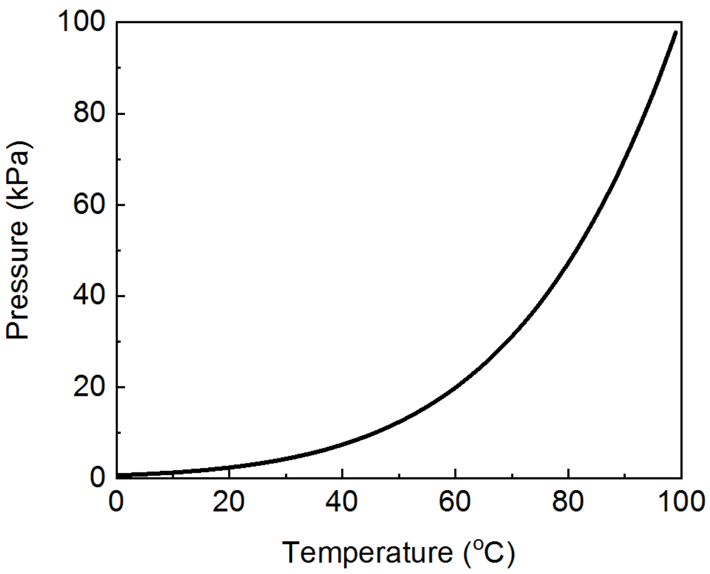

Supplementary Figure 10 Vapor pressure of water as a function of temperature.

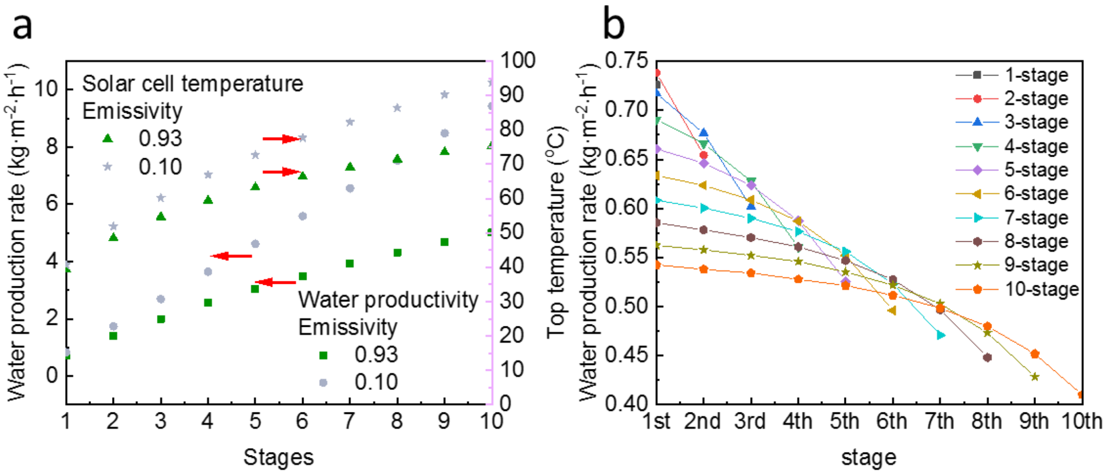

Supplementary Figure 11 Thermal modeling results of the PV-MD device. **a** The water production rate and solar cell temperature as a function of the number of the stage, **b** water production rate of each stage in the dead-end mode PV-MD with 1 to 10 stages structure. The solar absorptance and emissivity of the solar cell was set to 0.10 and 0.93, respectively.

Supplementary Note 3

A simplified thermal model is established for the PV-MD device based on the following assumptions: (1) the heat loss from side surfaces of the device is negligible owing to the low conductivity of the PU foam; (2) the latent heat of the water evaporation is constant and the sensible heat is negligible;(3) clean water is used in the model (4) the temperature at the bottom of the device is equal to the temperature of the ambient environment, which is constant at 25 ^{o}C.

The energy balance between different stages can be calculated using the following equation:

$$\alpha \times q_s - q_e - E = J_{bottom} \times L_v + \frac{k \times \Delta T_{bottom}}{\delta} = \cdots\cdots = J_i \times L_v + \frac{k \times \Delta T_i}{\delta} = \cdots\cdots = J_{top} \times L_v + \frac{k \times \Delta T_{top}}{\delta} \quad (1)$$

Where q_s refers to the incoming solar energy, α is the solar absorptance of solar cell, q_e is the captured solar energy that is converted to electricity, E is the thermal radiation energy loss, J_{bottom}, J_i and J_{top} are the water production rate in the bottom, i^{th} and top stage, L_v is the latent heat of the water evaporation and is assumed to be constant in this case, k and δ are the thermal conductivity and thickness of the hydrophobic porous membrane, ΔT_{bottom}, ΔT_i and ΔT_{top} are the temperature difference between the evaporation layer and condensation layer in the bottom, i^{th} and top stage.

The thermal radiation energy loss can be calculated in the following equation:

$$E = \varepsilon\sigma(T_{Cell}^4 - T_0^4) \quad (2)$$

Where ε is the emissivity of the material, σ is the Stefan-Bolzmann constant, T_{Cell} is the solar cell temperature, and T_0 is the temperature of its surroundings.

The temperature polarization effect in each evaporation layer and condensation layer should be taken into account owing to the low thermal conductivity of water. The heat into the device coming from the AlN layer in the first stage of the PV-MD device is $(\alpha \times q_s - q_e - E)$. The heat flux from the evaporation layer in the first stage to the condensation layer in the last stage should be close to this value because the sensible heat of the water can be reasonably neglected in this case. Given the porosity of the QGF membrane (94%), 94% volume of the evaporation layer is occupied by water, and therefore it's reasonable to directly use the thermal conductivity of water for the evaporation layer. It should be mentioned that the temperature polarization in condensation layer and evaporation layers are the same as they are made of the same materials with the same structure.

As a result, the temperature gradient in the evaporation layer and condensation layer can be calculated using the following equation:

$$\Delta T_e = \Delta T_c = \frac{\delta_e \times (\alpha \times q_s - q_e - E)}{k_e} \quad (3)$$

Where ΔT_e and ΔT_c are the polarization temperature of the evaporation layer and condensation layer, δ_e and k_e are the thickness and thermal conductivity of the evaporation layer.

For the water production rate of the ith stage, it can be calculated by the following equation[1]:

$$J_i = C_m \times (P_{ie} - P_{ic}) \quad (4)$$

Where C_m refers to the membrane coefficient of the hydrophobic membrane, which is constant in this case for different stages, P_{ie} and P_{ic} are the saturated vapor pressure of the evaporation layer and condensation layer in the ith stage. The vapor pressure (P) can be calculated by Antoine equation[2]:

$$lnP = Z - \frac{B}{D+T} \quad (5)$$

Where T is temperature and Z, B and D are constants (18.41 for Z, 3903.66 for B and 231.60 for D). The calculated result of P as a function of T for water is shown in Fig. S10. As seen, with an increasing temperature, the vapor pressure of water increases sharply, indicating that a higher vapor pressure difference can be achieved with the same temperature difference (i.e., $T_{ie} - T_{ic}$) when the temperature is higher.

A LabView module was programed and used to calculate the temperature of top surface of a PV-MD device with a given stage number, preset solar absorptance and thermal emissivity of the solar cell, based on the above described thermal model. The temperature of each conduction layer and the clean water production rate of each stage of the device with 3 to 10 stages are calculated in this work using an iterative method.

Taking a 3-stage device as an example, T_{3e} is firstly assigned a number that a little higher than room temperature (*e.g.*, 25.01ºC) and the water production rate in the 3rd stage is then calculated by Supplementary Equation 4 and 5 since T_{3c} is equal to room temperature. The total energy input into the 3rd stage (q_3) is calculated by Supplementary Equation 1, based on which the polarization temperature (ΔT_c and ΔT_e) can be calculated by Supplementary Equation 3 for the 3rd stage.

The T_{2c} is subsequently obtained by adding the polarization temperature ($\Delta T_c + \Delta T_e$) to T_{3e}. Now, assuming T_{2e} is a bit higher than T_{2c} (*e.g.*, T_{2c} + 0.01), Supplementary Equation 1 is then used to calculate the energy input into the 2nd stage (q_2). If q_2 is not equal to q_3, T_{2e} is increased by 0.01 and q_2 is calculated again until q_2 is equal to q_3. Thereafter, T_{1e} is similarly calculated and the temperature of the top surface of the solar cell can be calculated by adding ΔT_e to T_{1e}. After obtaining the top surface temperature, the power of the heat loss *via* thermal radiation can be calculated and subsequently the total heat flow into the MSMD device ($\alpha \times q_s - q_e - E$) is calculated based on Supplementary Equation 1. If ($\alpha \times q_s - q_e - E$) is not equal to q_3, T_{3e} is increased by 0.01 and the whole computation cycle for all parameters is performed again until ($\alpha \times q_s - q_e - E$) is reasonably close to q_3 (i.e., $|(\alpha \times q_s - q_e - E)-q_3|<1$) where the values of all parameters are obtained.

Supplementary Figure 11a presents the simulated water production rate and solar cell temperature as a function of the total stages of PV-MD device. As seen, the PV-MD device shows an increased water production performance along with increasing number of total stages, which agrees well with the experimental results of this work and others[3].

However, our model results show that the solar cell temperature increases monotonically as the number of the total stages increases. It is also indicated that the emissivity of the solar cell can significantly affect the water production performance of the device. The PV-MD with a high emissivity (0.93) shows a considerably poorer water production performance than the one with low emissivity (0.10). The difference is widened as the number of stages increases (Supplementary Figure 11a). For example, with the number of stages is greater than 7, the water production rate of the device with an emissivity of 0.93 is >40% less than that with an emissivity of 0.10.

The difference can be explained by thermal radiation at the solar cell. As the number of the stages increases, the solar cell temperature increases accordingly which results in a higher thermal radiation energy loss as shown in Supplementary Equation 2. However, our results imply that reducing the emissivity of the solar cell can be considered as an effective strategy to further improve the water production performance of PV-MD device.

Supplementary Figure 11b presents the water production rate of each stage in the PV-MD device with a high emissivity of 0.93. Clearly, the water production rate of each stage decreases from top to bottom stages in such device, although the total water production rate by the entire device increases monotonically. This follows that the effectiveness of lower stages would be lower than upper stages within the same device. Given the same fabrication cost of each stage, there ought to be a break-even total number of stages for such a device beyond which further increase in the number of stages would not be cost-effective.

Furthermore, from the solar cell point of view, both of its lifetime and electricity generation efficiency will be adversely affected as the cell temperature increases as a result of the increased stage number.

Based on the above discussions, the number of stages in a PV-MD device should be limited to a certain value due to holistic considerations of device fabrication cost, solar cell efficiency, and water production rate.

Supplementary Table 1 Performances of solar still reported in literature.

Year	Evaporation rate/kg·m^{-2}·h^{-1}	Efficiency		Solar intensity/kW·m^{-2}
		Steam generation	Electricity generation	
This work	1.79 (collection rate)	126%	11.55%	1
	2.78 (collection rate)	195%	0	1
2017[4]	1.15	75%	~0.1%	1
2018[5]	34.8	72%	1.23%	30
2018[6]	1.39	90%	~0.01%	1
2018[3]	3.27	-	0	0.9 (electrical heating)
2018[7]	1.02	72%	0	1
2018[8]	1.59	85%	0	1
2018[9]	1.12	81%	0	1
2018[10]	5.10	80%	0	4
2018[11]	2.50	95%	0	1
2018[12]	1.30	72%	0	1
2018[13]	11.8	85%	0	10
2018[14]	1.24	83%	0	1
2018[15]	2.04	99%	0	1
2018[16]	1.32	88%	0	1
2018[17]	6.60	92%	0	5
2018[18]	1.08	74%	0	1
2017[19]	1.13	78%	0	1
2017[20]	1.70	85%	0	1
2017[21]	3.00	66%	0	3
2017[22]	1.00	82%	0	1
2017[23]	12.1	87%	0	10
2017[24]	1.18	76%	0	1
2017[25]	1.55	91%	0	1
2017[26]	0.90	65%	0	1
2017[27]	11.80	85%	0	10
2017[28]	3.46	72%	0	3
2017[29]	1.25	86%	0	1
2017[30]	14.02	83%	0	12
2017[31]	11.22	81%	0	10
2017[32]	1.48	78%	0	1
2017[33]	1.32	84%	0	1
2017[34]	1.62	83%	0	1
2016[35]	1.45	80%	0	1
2016[36]	13.50	64%	0	12
2016[37]	11.80	83%	0	10
2016[38]	5.20	90%	0	4
2016[39]	1.25	82%	0	1
2016[40]	5.70	90%	0	4
2015[41]	1.50	80%	0	1
2015[42]	5.60	78%	0	4.5
2015[43]	0.92	58%	0	1
2014[44]	13.20	85%	0	10

Supplementary Note 4

Broadly speaking, water and electricity can also be simultaneously produced by such hybrid systems as photovoltaic-reverse osmosis (PV-RO) and organic Rankine cycle-photovoltaic reverse osmosis (ORC-PV RO).[45,46] Although these systems can produce clean water with a much higher water production rate of around ~250 m^3/day, they typically have a much higher barrier-to-entry with a very high up-front capital expenditure investment, which limits their applications to de-centralized water production at small to medium scales. Besides, electricity produced in these systems is typically fully consumed by the RO processes. In comparison, all of the electricity generated in the PV-MD system is available to feed into commercial grid plus free clean water production.

Due to various reasons, near half of the world population is living near the sea (10.1371/journal.pone.0118571). One possible and emerging solution for PV plants is to build them in the sea land. For example, the world's biggest floating solar farm has just been put in operation in Queen Elizabeth II reservoir in UK (https://www.ciel-et-terre.net/essential_grid/floating-solar-system-qe2-633765-kwp/) and similar floating solar farms are going to be built on seawater at various places in the world (https://www.popularmechanics.com/science/energy/a18197171/netherlands-to-build-a-solar-farm-that-will-float-in-the-ocean/). If the PV-MD can be used in these projects, significant amount of clean water can be produced from seawater without electricity consumption, unlike conventional desalination processes (e.g., RO, MED, MSF).

In addition, almost all the deserts in Middle East, North Africa, Southwest Africa, West Australia, and South America are close to the sea and the majority of the human population in these areas lives near the seashore. Therefore, the access to the seawater is not a big problem in these deserts areas for the PV-MD.

Actually, only in middle Asia and North America, the deserts are located in land-locked areas. Even in some of these areas, salty water/wastewater are massively produced by industry. For example, in the north and west part of China where there is a very high fresh water stress, coal-burning power plants produce a lot of brine wastewater (> 2 billion m^3/year). The device reported in this work can be used for water recovery from the brine wastewater to contribute to water reuse in these regions where solar irradiation is abundant and fresh water is very scarce.

Supplementary references

1 Alkhudhiri, A., Darwish, N. & Hilal, N. Membrane distillation: A comprehensive review. *Desalination* **287**, 2-18 (2012).
2 Smallwood, I. *Handbook of organic solvent properties*. (Butterworth-Heinemann, 2012).
3 Chiavazzo, E., Morciano, M., Viglino, F., Fasano, M. & Asinari, P. Passive solar high-yield seawater desalination by modular and low-cost distillation. *Nature Sustainability* **1**, 763-772 (2018).
4 Yang, P. *et al.* Solar-driven simultaneous steam production and electricity generation from salinity. *Energy Environ. Sci.* **10**, 1923-1927 (2017).
5 Li, X. *et al.* Storage and recycling of interfacial solar steam enthalpy. *Joule* **2**, 2477-2484 (2018).
6 Zhu, L., Gao, M., Peh, C. K. N., Wang, X. & Ho, G. W. Self-contained monolithic carbon sponges for solar-driven interfacial water evaporation distillation and electricity generation. *Adv. Energy Mater.* **8**, 1702149 (2018).

7 Xue, G. *et al.* Highly efficient water harvesting with optimized solar thermal membrane distillation device. *Glob. Challenges* **2**, 1800001 (2018).
8 Hong, S. *et al.* Nature-Inspired, 3D Origami Solar Steam Generator toward Near Full Utilization of Solar Energy. *ACS Appl. Mater. Interfaces* **10**, 28517-28524 (2018).
9 Chen, Q. *et al.* A durable monolithic polymer foam for efficient solar steam generation. *Chem. Sci.* **9**, 623-628 (2018).
10 Chen, C. *et al.* Dual functional asymmetric plasmonic structures for solar water purification and pollution detection. *Nano Energy* **51**, 451-456 (2018).
11 Zhou, X., Zhao, F., Guo, Y., Zhang, Y. & Yu, G. A hydrogel-based antifouling solar evaporator for highly efficient water desalination. *Energy Environ. Sci.* **11**, 1985-1992 (2018).
12 Xu, W. *et al.* Flexible and salt resistant janus absorbers by electrospinning for stable and efficient solar desalination. *Adv. Energy Mater.* **8**, 1702884 (2018).
13 Zhu, M. *et al.* Plasmonic wood for high-efficiency solar steam generation. *Adv. Energy Mater.* **8**, 1701028 (2018).
14 Chang, J. *et al.* Solar-assisted fast cleanup of heavy oil spills using a photothermal sponge. *J. Mater. Chem. A* **6**, 9192-9199 (2018).
15 Shi, Y. *et al.* A 3D photothermal structure toward improved energy efficiency in solar steam generation. *Joule* **2**, 1171-1186 (2018).
16 Shi, Y. *et al.* A robust $CuCr_2O_4/SiO_2$ composite photothermal material with underwater black property and extremely high thermal stability for solar-driven water evaporation. *Adv. Sustainable Syst.* **2**, 1700145 (2018).
17 Yang, X. *et al.* An ultrathin flexible 2D membrane based on single-walled nanotube-MoS_2 hybrid film for high-performance solar steam generation. *Adv. Funct. Mater.* **28**, 1704505 (2018).
18 Liu, H. *et al.* High-performance solar steam device with layered channels: artificial tree with a reversed design. *Adv. Energy Mater.* **8**, 1701616 (2018).
19 Jiang, Q. *et al.* Polydopamine-filled bacterial nanocellulose as a biodegradable interfacial photothermal evaporator for highly efficient solar steam generation. *J. Mater. Chem. A* **5**, 18397-18402 (2017).
20 Li, X. *et al.* Three-dimensional artificial transpiration for efficient solar waste-water treatment. *Natl. Sci. Rev.* **5**, 70-77 (2017).
21 Fu, Y. *et al.* Accessible graphene aerogel for efficiently harvesting solar energy. *ACS Sustainable Chem. Eng.* **5**, 4665-4671 (2017).
22 Wang, H., Miao, L. & Tanemura, S. Morphology control of Ag polyhedron nanoparticles for cost-effective and fast solar steam generation. *Solar RRL* **1**, 1600023 (2017).
23 Jia, C. *et al.* Rich mesostructures derived from natural woods for solar steam generation. *Joule* **1**, 588-599 (2017).
24 Liu, C. *et al.* High-performance large-scale solar steam generation with nanolayers of reusable biomimetic nanoparticles. *Adv. Sustainable Syst.* **1**, 1600013 (2017).
25 Gao, X. *et al.* Synthesis of hierarchical graphdiyne-based architecture for efficient solar steam generation. *Chem. Mater.* **29**, 5777-5781 (2017).
26 Wang, G. *et al.* Reduced graphene oxide–polyurethane nanocomposite foam as a reusable photoreceiver for efficient solar steam generation. *Chem. Mater.* **29**, 5629-5635 (2017).
27 Wang, X., He, Y., Liu, X., Cheng, G. & Zhu, J. Solar steam generation through bio-inspired interface heating of broadband-absorbing plasmonic membranes. *Appl. Energy* **195**, 414-425 (2017).
28 Xue, G. *et al.* Robust and low-cost flame-treated wood for high-performance solar steam generation. *ACS Appl. Mater. Interfaces* **9**, 15052-15057 (2017).

29 Li, Y. *et al.* 3D-printed, all-in-one evaporator for high-efficiency solar steam generation under 1 sun illumination. *Adv. Mater* **29**, 1700981 (2017).

30 Liu, K. K. *et al.* Wood-graphene oxide composite for highly efficient solar steam generation and desalination. *ACS Appl. Mater. Interfaces* **9**, 7675-7681 (2017).

31 Chen, C. *et al.* Highly flexible and efficient solar steam generation device. *Adv. Mater* **29**, 1701756 (2017).

32 Xu, N. *et al.* Mushrooms as efficient solar steam-generation devices. *Adv. Mater* **29**, 1606762 (2017).

33 Li, R., Zhang, L., Shi, L. & Wang, P. MXene Ti_3C_2: an effective 2D light-to-heat conversion material. *ACS nano* **11**, 3752-3759 (2017).

34 Hu, X. *et al.* Tailoring graphene oxide-based aerogels for efficient solar steam generation under one sun. *Adv. Mater* **29**, 1604031 (2017).

35 Li, X. *et al.* Graphene oxide-based efficient and scalable solar desalination under one sun with a confined 2D water path. *Proc. Natl. Acad. Sci. USA* **113**, 13953-13958 (2016).

36 Ni, G. *et al.* Steam generation under one sun enabled by a floating structure with thermal concentration. *Nat. Energy* **1**, 16126 (2016).

37 Jiang, Q. *et al.* Bilayered biofoam for highly efficient solar steam generation. *Adv. Mater* **28**, 9400-9407 (2016).

38 Zhou, L. *et al.* Self-assembly of highly efficient, broadband plasmonic absorbers for solar steam generation. *Sci. Adv.* **2**, 1501227 (2016).

39 Wang, Y., Zhang, L. & Wang, P. Self-floating carbon nanotube membrane on macroporous silica substrate for highly efficient solar-driven interfacial water evaporation. *ACS Sustainable Chem. Eng.* **4**, 1223-1230 (2016).

40 Zhou, L. *et al.* 3D self-assembly of aluminium nanoparticles for plasmon-enhanced solar desalination. *Nat. Photonics* **10**, 393-398 (2016).

41 Ito, Y. *et al.* Multifunctional porous graphene for high-efficiency steam generation by heat localization. *Adv. Mater* **27**, 4302-4307 (2015).

42 Liu, Y. *et al.* A bioinspired, reusable, paper-based system for high-performance large-scale evaporation. *Adv. Mater* **27**, 2768-2774 (2015).

43 Zhang, L., Tang, B., Wu, J., Li, R. & Wang, P. Hydrophobic light-to-heat conversion membranes with self-healing ability for interfacial solar heating. *Adv. Mater* **27**, 4889-4894 (2015).

44 Ghasemi, H. *et al.* Solar steam generation by heat localization. *Nat. Commun* **5**, 5449-5455 (2014).

45 Karellas, S., Terzis, K. & Manolakos, D. Investigation of an autonomous hybrid solar thermal ORC–PV RO desalination system. The Chalki island case. *Renewable Energy* **36**, 583-590 (2011).

46 Manolakos, D., Mohamed, E. S., Karagiannis, I. & Papadakis, G. Technical and economic comparison between PV-RO system and RO-Solar Rankine system. Case study: Thirasia island. *Desalination* **221**, 37-46 (2008).
